# Impact of Temperature Variation on Acute Myocardial Infarction in Karachi, Pakistan

**DOI:** 10.7759/cureus.5910

**Published:** 2019-10-15

**Authors:** Sanam Khowaja, Musa Karim, Maham Zahid, Annam Zahid, Salik Ahmed, Khawar Kazmi, Syed Z Jamal

**Affiliations:** 1 Adult Cardiology, National Institute of Cardiovascular Diseases, Karachi, PAK; 2 Miscellaneous, National Institute of Cardiovascular Diseases, Karachi, PAK; 3 Medicine, Ziauddin Medical University, Karachi, PAK; 4 Preventive Cardiology, National Institute of Cardiovascular Diseases, Karachi, PAK; 5 Electrophysiology, National Institute of Cardiovascular Diseases, Karachi, PAK

**Keywords:** seasonal, percutaneous coronary intervention, temperature, myocardial infarction

## Abstract

Introduction

Environmental triggers of acute myocardial infarction (AMI) have gained mounting evidence from various geographies of the world. However, due to geographic variations in seasonal temperature and other metrological parameters, it is difficult to generalize the findings in one population to another population with different climatic conditions. Therefore, the aim of this study was to assess the relationship between meteorological parameters and the number of primary percutaneous coronary intervention (PCI) procedures for AMI at a tertiary care cardiac hospital in Karachi, Pakistan.

Methods

For this cross-sectional study, data was obtained on the number of primary PCI procedures conducted at the National Institute of Cardiovascular Diseases (NICVD) Karachi, Pakistan during 1^st^ June 2016 to 31^st^ May 2018. Daily meteorological data of the Karachi region for the same period was obtained from the Pakistan Meteorological Department. It consists of temperature, atmospheric pressure, and relative humidity. Based on the weather conditions of Karachi, the data was divided into two seasons; summer (April to October) and winter (November to March). Multiple linear regression analysis was performed taken the number of primary PCI performed as regressand and time trend, average temperature, temperature variation, and relative humidity as regressors.

Results

A total of 115,494 hospital admissions were recorded during the study period out of which rate of primary PCI was 10.5% (12,107). A negative relationship between average temperature and number of primary PCI was observed with standardized regression coefficients of -0.13 (*p *< 0.001) on the overall regression model. A similar significant negative relationship of average temperature was observed on the regression model for the cold season with standardized regression coefficients of -0.17 (*p *< 0.001). While no such relationship was observed for the warm season.

Conclusion

The average daily temperature was found to be negatively related to the number of primary PCI. Subgroup analysis revealed that the average daily temperature had a significant negative relationship with the number of primary PCI in the cold season; however, no such impact was observed in the warm season.

## Introduction

The burden of cardiovascular diseases (CVDs) remains the global challenge, with around 422.7 million cases and 17.92 million death attributed to it, and ischemic heart disease (IHD) is the leading cause of health losses, in terms of chronic disabilities and premature mortalities, for almost all parts of the world [1]. Rigorous implementation of various preventive strategies such as the use of cardio-protective diet and medications, adaptation of active lifestyle, and ongoing tobacco cessation campaigns as well as innovations and developments in the patient care results in a sustainable reduction in mortality rate associated with coronary heart diseases (CHD) in high-income countries [2]. However, 80% of the mortalities associated with cardiovascular diseases (CVDs) are still from low and middle-income countries [3].

Dominating causes of hospital admission of patients with CVDs include coronary heart disease, heart failure and cardiomyopathy, stroke, peripheral vascular disease, and others. Acute myocardial infarction (AMI) is the lethal manifestation of coronary artery disease associated with increased morbidity and mortality [4]. Coronary thrombosis, a sudden restriction or decrease in blood flow due to the rupture of atherosclerotic plaque, is the leading precipitant of AMI [5]. Factors modulating AMI is a subject of prime interest for clinicians these days because it could help us to prevent AMI and allocate our health resources in the right direction. Quantification of the exact triggering factor of AMI cannot be possible for every case in clinical practice [5]. However, various epidemiological and clinical studies have identified several associated triggering factors for AMI, which include, heavy physical exertion, emotional liability, drug abuse, sexual activities, and diet [5-8].

A number of environmental factors play an important part in its prevalence, both geographical and environmental. Traditionally AMI is considered a disease of hot and humid weather. Moreover, in the past few decades, environmental triggers of AMI have gained mounting evidence from various geographies of the world [5,7-14]. Low temperature or cold weather was found to be a convincing risk factor for the incidence of AMI [5,7]. However, due to geographic variations in seasonal temperature and other metrological parameters, it is difficult to generalize the findings in one population to another population with different climatic conditions. Hence, data are available on negating the association of atmospheric temperature with an increasing incidence of AMI [15].

With these compelling evidence of the association between climatic conditions and risk of AMI, climatic conditions and variations need to be addressed appropriately in our risk reduction strategies of AMI [5]. Available data are mostly from western countries and very limited data are available for South Asian countries, especially Pakistan. Climatic conditions are reasonably different on our side of the equator, and therefore, it is important to analyze and understand the local and regional scenarios. The aim of this study was to assess the relationship between meteorological parameters and the number of primary percutaneous coronary intervention (PCI) procedures for AMI performed at a tertiary care cardiac hospital in Karachi, Pakistan.

## Materials and methods

This study was conducted at the National Institute of Cardiovascular Diseases (NICVD), Karachi, Pakistan, the largest cardiac care center in Pakistan. For this study, the number of primary PCI procedures performed at NICVD every day was taken as the surrogate variable for the incidence rate of AMI on a given day. Data on the number of primary PCI procedures performed daily for the duration of 1^st^ June 2016 to 31^st^ May 2018 was obtained. Daily meteorological data of the Karachi region for the same period, i.e. 1^st^ June 2016 to 31^st^ May 2018, was obtained from the Pakistan Meteorological Department (PMD). Meteorological data consist of temperature (maximum, minimum, and average; ºC), atmospheric pressure sea level (hPa/gpm) at 0000 coordinated universal time (UTC) and relative humidity (%) at 1200 UTC.

Diagnosis and management of AMI were carried out as per the ACCF/AHA/SCAI (American College of Cardiology Foundation/American Heart Association/Society for Cardiovascular Angiography and Interventions) guidelines [16]. AMI or ST-segment elevation myocardial infarction (STEMI) was defined as a patient presenting with substernal chest pain for more than 20 minutes described as a squeezing or constricting sensation with frequent radiation to the left arm and met any two of the electrocardiographic (ECG) criteria, a) new ST elevation at the J-point in at least two contiguous leads with the cut-off points: ≥2 mm in men or ≥1.5 mm in women in leads V2-V3, b) new ST elevation of ≥1 mm in contiguous chest leads, c) new ST elevation of ≥1 mm in contiguous limb leads, or d) the typical rise of cardiac troponin one value above the upper limit of normal range.

Based on the weather condition of Karachi region, the Gregorian calendar was divided into two seasons, warm season (April to October) and cold season (November). Temperature variation was taken as the difference between the maximum and minimum temperature for the day. Variables at disposal for the analysis are the number of primary PCI performed as regressand (dependent variable) and time trend, temperature variation, average temperature, atmospheric pressure, and relative humidity as regressors (explanatory variables). The hottest days in summer, days with an abnormally high temperature, were identified using the criteria of maximum temperature above the upper limit of 90% confidence interval of the mean maximum temperature (mean + 1.96(standard deviation)) of the month. Similarly, the coldest days in winter, days with an abnormally low temperature, were identified using the criteria of minimum temperature below the lower limit of 90% confidence interval of the mean minimum temperature (mean - 1.96(standard deviation)) of the month. The heat index was calculated using average temperature and the formula used for the calculation of heat index is defined elsewhere [17].

Data analyses were performed using R version 3.5.1 (The R Foundation for Statistical Computing) and IBM SPSS Statistics for Windows, Version 21.0. (IBM Corp., Armonk, NY, US). Multiple linear regression analysis was performed to analyze the relationship between regressand and regressors. Considering the time-series behavior of data a trend variable, starting with the value of one for the first day in the analysis period and increased by one for every successive day, was introduced as a regressor.

Six important assumptions of multiple linear regression were tested in order to generate a valid and reliable model and interpretation.

• Assumption 1 - Linear relationship between regressand and regressors: as a preliminary step linear relationship between the number of primary PCI procedures and individual explanatory variables were assessed by computing correlation coefficient, scatter plots, and time series plots.

• Assumption 2 - Multicollinearity among the explanatory variables was assessed by computing VIF with a cutoff value of less than 10 and a tolerance score with a cutoff value of more than 0.2.

• Assumption 3 - Autocorrelation in residuals: Durbin-Watson test was applied to test the hypothesis of autocorrelation or independent residuals of the regression model and as a rule of thumb test statistic value between 1.5 and 2.5 was considered as evidence for no serial correlation or autocorrelation in residuals.

• Assumption 4 - Homoscedasticity of residuals: Under the assumption, residuals of the regression model should be homoscedastic or should have constant variance, this assumption was visually tested for any apparent pattern on scatter plot between standardized residuals and standardized predicted values of the model.

• Assumption 5 - Normally distributed residuals: The fifth assumption was tested visually by assessing normal probability (P-P) plot and by applying the Kolmogorov-Smirnov (KS) test.

• Assumption 6 - No bias due to influential cases or extreme values: This assumption was tested by computing Cook’s distance, and distance values above one were classified as potential outlier cases.

For all the univariate and multivariate analysis, *p*-value ≤ 0.05 was taken as criteria for statistical significance.

## Results

**Figure 1:**
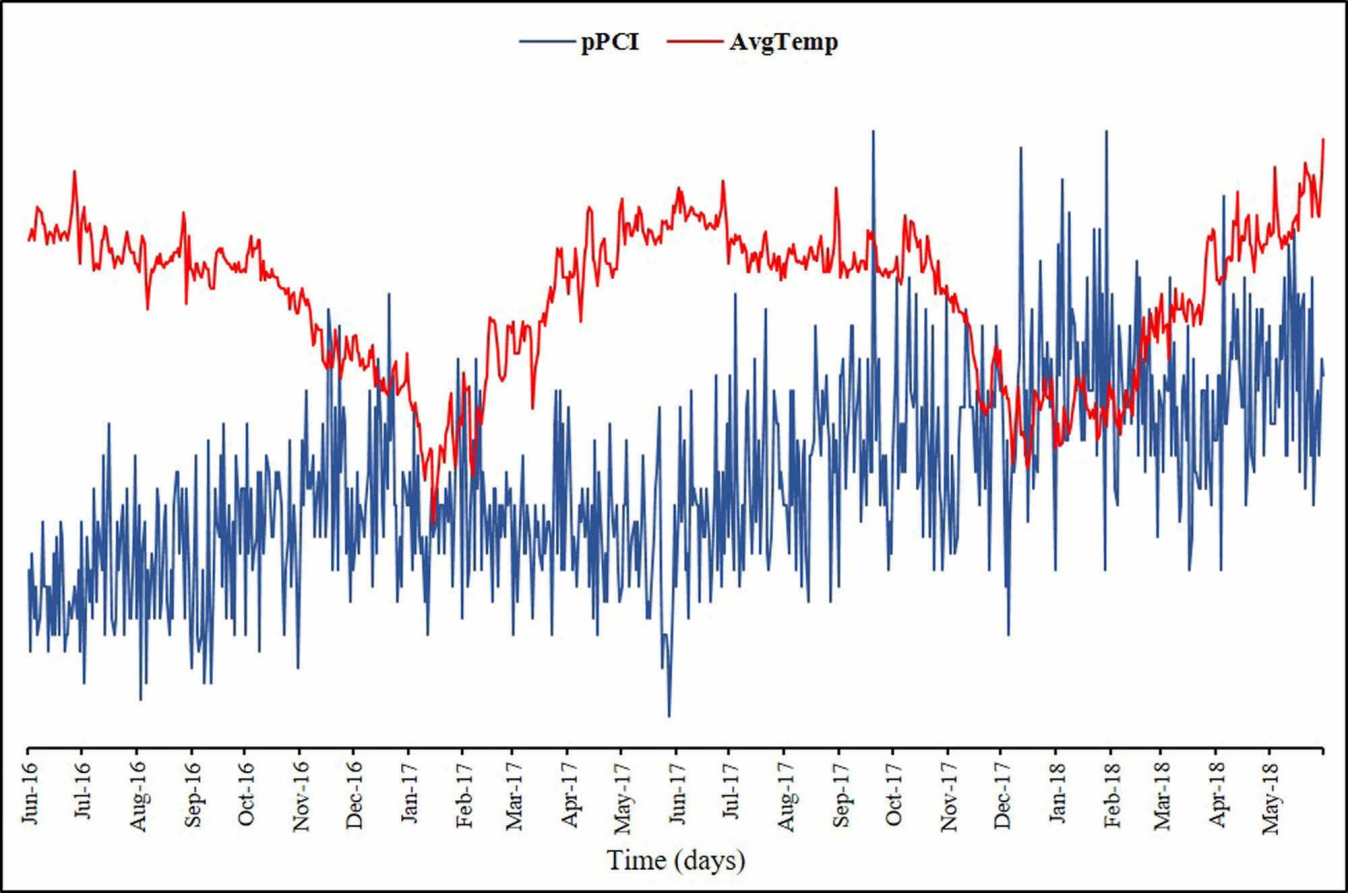
Temporal trend of the number of primary PCI procedures and average temperature pPCI, primary percutaneous coronary intervention, AvgTemp, average temperature

A total of 115,494 hospital admissions were recorded during the study period, out of which the rate of primary PCI procedure was 10.5% (12,107). An increasing trend in the number of primary PCI was observed over time. Figure 1 shows a temporal trend of the number of primary PCI procedures performed.

**Figure 2:**
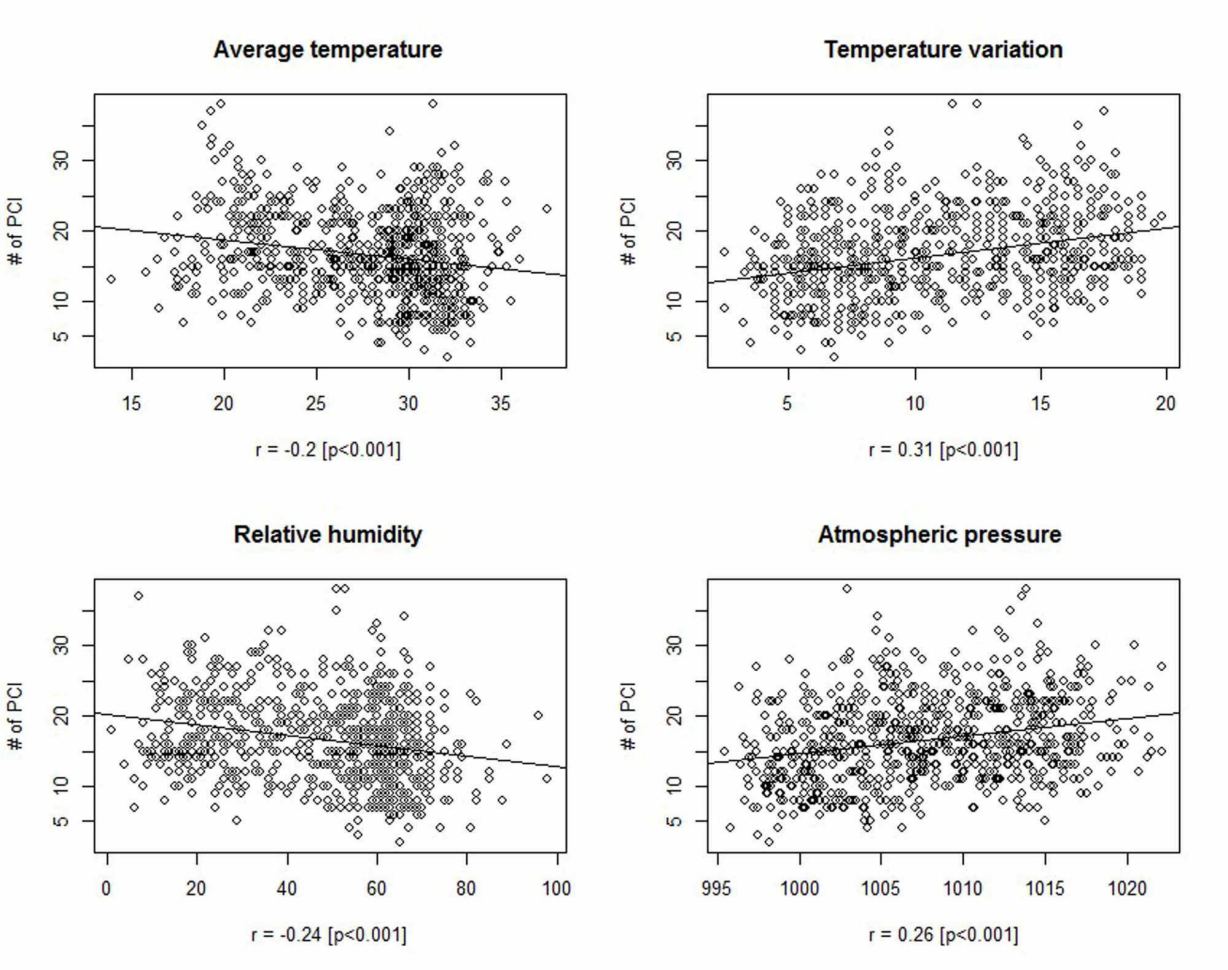
Scatter plot showing a linear relationship between regressand and regressors r = correlation

Figure 2 evidently shows the linear relationship between the number of primary PCI procedures, the regressand, and the regressors. A negative relationship of the number of primary PCI procedures was observed with average daily temperature and relative humidity with a correlation coefficient of -0.20 (*p *< 0.001) and -0.24 (*p *< 0.001) respectively. A positive relationship between the number of primary PCI procedures and temperature variation and atmospheric pressure (hPa/gpm) was observed with a correlation coefficient of 0.31 (*p *< 0.001) and 0.26 (*p *< 0.001), respectively. 

The average number of primary PCI procedures performed on the same day and the following day of days with extreme temperatures is presented in Table 1. No statistically significant differences were observed in the mean number of primary PCI procedures for days with extreme and normal temperatures in both seasons.

**Table 1:** The average number of primary PCI procedures performed on the same day and following day of days with extreme temperatures PCI, percutaneous coronary intervention; SD, standard deviation

	Same day			Following day		
	Base	Mean ± SD	p-value	Count	Mean ± SD	p-value
Cold Season						
Coldest	16	17.06 ± 6.20	0.41	16	18.69 ± 6.24	0.717
Normal	286	18.26 ± 5.62		286	18.15 ± 5.71	
Warm Season						
Hottest	34	16.32 ± 6.94	0.382	34	16.00 ± 5.18	0.573
Normal	394	15.37 ± 6.01		394	15.39 ± 6.10	

**Figure 3:**
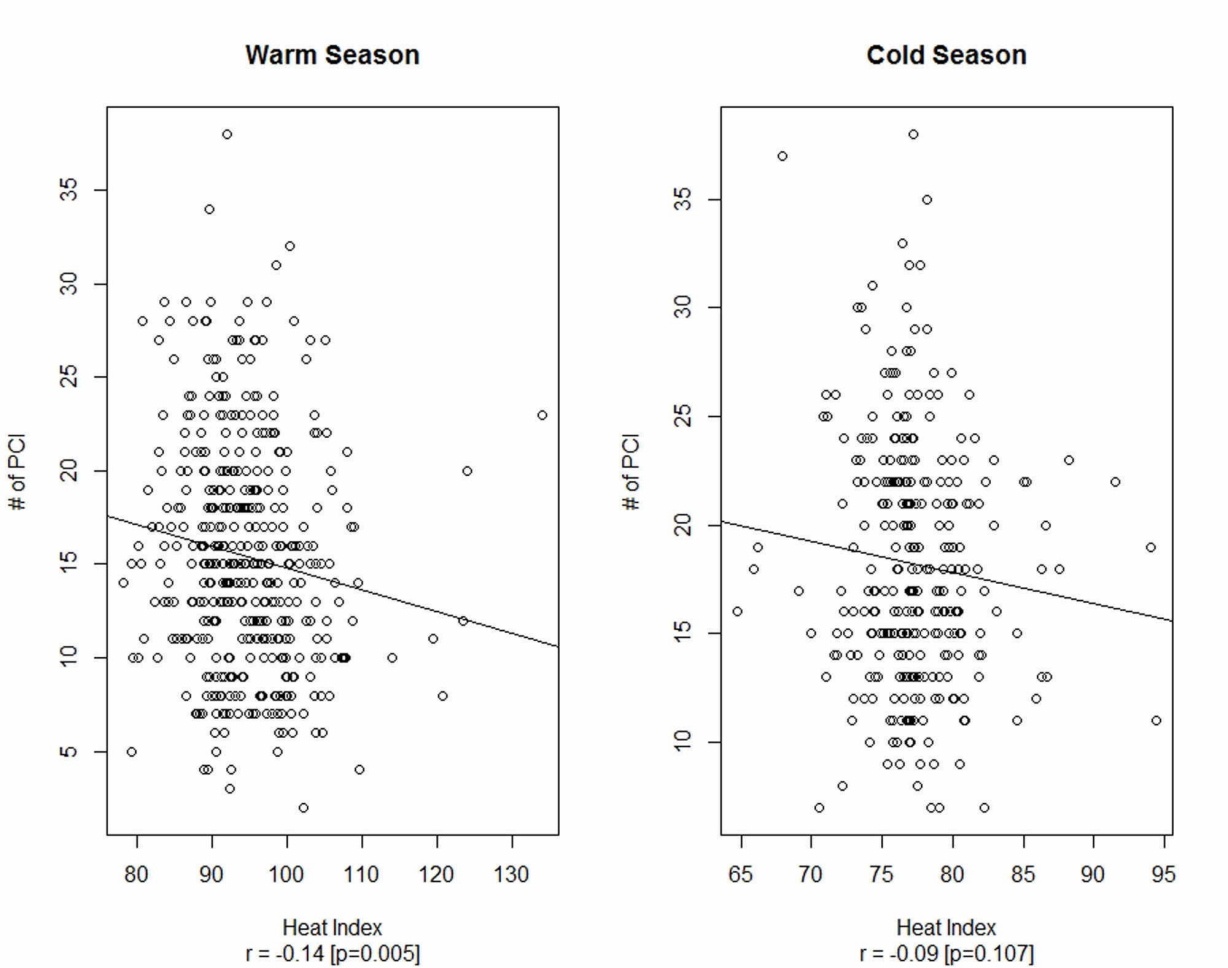
The relationship between heat index and number of primary PCI in the warm and cold seasons PCI, percutaneous coronary intervention

The relationship between the heat index and the number of primary PCI procedures in the warm and cold season is presented in Figure 3. A very weak correlation between heat index and the number of primary PCI procedures was observed.

Multiple linear regression analysis was performed with the number of primary PCI procedures performed as regressand (dependent variable) and five regressors namely time trend, temperature variation, average temperature, atmospheric pressure, and relative humidity. Atmospheric pressure was found to be strongly correlated with average temperature and temperature variation, with a correlation coefficient of -0.817 and 0.739, respectively, and tolerance value was on borderline (0.195) with VIF of 5.13 (<10); therefore, atmospheric pressure variable was removed from the regression model. VIF for the four regressors ranged from 1.13 to 2.32 and tolerance ranged from 0.43 to 0.88, indicating no evidence of multicollinearity among the explanatory variables. Therefore, the second assumption, multicollinearity, of the linear regression analysis was not violated. Durbin-Watson test statistic value of 1.85 was found within the acceptable range of 1.5 to 2.5; hence, third assumption, no autocorrelation in residuals, was confirmed. No apparent patterns were observed on the scatter plot of standardized residuals and standardized predicted values of the regression model; therefore, the residuals of our regression model are homoscedastic as per the fourth assumption. Even though a majority of the points on our P-P plot of residuals lie near the diagonal line but KS test of normality rejected with *p *= 0.004, therefore our regression model failed to comply the fifth assumption of normally distributed residuals. Finally, Cook’s distance value ranged over 0 to 0.026; hence, our data comply with the sixth assumption of no influential cases or extreme values. Status of the six assumptions for the multiple linear regression models based on overall data and data for the warm and cold seasons are presented in Table 2.

**Table 2:** Six assumptions for the linear regression models based on overall data and data for warm and cold seasons KS, Kolmogorov-Smirnov; P-P, normal probability

Assumption	Criteria for the assumption to met	Regression Models		
		Overall	Warm	Cold
1 - Linearity of relationship	Visual evidence of linear relationship on scatter plot	Met	Met	Met
2 - No multicollinearity among regressors	Variance inflation factor less than 10 and tolerance score more than 0.2	Met	Met	Met
3 - No autocorrelation in residuals	Durbin-Watson test statistics within 1.5 to 2.5 range	Met	Met	Met
4 - Homoscedasticity of residuals	No apparent patterns on scatter plot of standardized residuals and predicted values	Met	Met	Met
5 - Normally distributed residuals	Majority of the points on P-P plot along the diagonal line or KS test p-value above 0.05	Not Met	Met	Met
6 - No extreme values / cases	No cases with Cook’s distance above 1 value	Met	Met	Met

An increasing trend in the number of primary PCI procedures was observed with standardized regression coefficients of 0.60 (*p *< 0.001) and it remains an important determinant regardless of seasons. A negative relationship between the average temperature and the number of primary PCI procedures was observed with standardized regression coefficients of -0.13 (*p *< 0.001), while temperature variation was found to be positively related with the number of primary PCI procedures with regression coefficients of 0.12 (*p *< 0.001) on the overall regression model. A similar significant negative relationship of average temperature and positive relationship of temperature variation was observed on regression model for the cold season with standardized regression coefficients of -0.17 (*p *< 0.001) and 0.16 (*p *< 0.001) respectively. While no such relationships of the number of procedures with average temperature and temperature variation were observed for the warm season. The multiple linear regression model coefficients for the overall warm and cold season models are presented in Table 3.

**Table 3:** Multiple linear regression model coefficients for the overall, warm season, and cold season models Regressand = number of primary PCI performed *Statistically significant at 5% level of significance β = unstandardized regression coefficients, Std. β = Standardized regression coefficients, p = p-value, R^2^ = coefficient of determination, Adj. R^2^ = adjusted coefficient of determination

Regressors	Total Model			Warm Model			Cold Model		
	β	Std. β	p	β	Std. β	p	β	Std. β	p
Constant	11.92	-	<0.001*	10.69	-	0.01*	14.59	-	<0.001*
Time trend	0.02	0.60	<0.001*	0.03	0.67	<0.001*	0.03	0.47	<0.001*
Average temperature	-0.17	-0.13	<0.001*	-0.12	-0.03	0.36	-0.29	-0.17	<0.001*
Temperature variation	0.17	0.12	<0.001*	0.11	0.06	0.22	0.34	0.16	<0.001*
Relative humidity	0.03	0.09	0.04*	0.01	0.02	0.65	0.02	0.06	0.25
R^2^ [Adj. R^2^]	0.414 [0.411]			0.460 [0.455]			0.237 [0.227]		

## Discussion

To the best of our knowledge, this study is the first of its kind in the local setup of Pakistan. In this study, we used the number of primary PCI procedures performed every day at the largest tertiary care cardiac center in Pakistan, as a surrogate variable for AMI and the temporal trend was addressed by introducing a trend variable in the analysis. We observed a negative relationship between daily numbers of primary PCI procedures and the average atmospheric temperature. This relationship is more evident in the cold season, while no such significant relationship exists between average daily temperature and the number of primary PCI procedures in the warm season. However, the correlation between temperature (heat index) and the number of primary PCI procedures for the cold season was weak (correlation = -0.14). 

A direct comparison of our study finding with previous studies is difficult owing to the differences and inconsistencies in the study design and statistical methods. On an aggregate level, we observed the same relationship between temperature and AMI, along with with the same seasonal variations, as the studies conducted in the past [5,9-14,18-20]. Among other metrological variables, temperature variations have a profound and consistent impact on the incidence of AMI, not only in its extreme states (both high and low) but also transient change in temperature can trigger acute myocardial infarction (AMI) [5]. The potential biological mechanism behind temperature variation-triggered AMI was explained as coronary plaque instability and myocardial ischemia precipitated by increased blood pressure and heart rate due to subsequent vasoconstriction after a rise in catecholamine levels due to the stimulation of cold receptors in the skin [18,21-24]. Furthermore, exposure to the cold temperature causes haemoconcentration and a decrease in plasma volume and an increase in diuresis and blood viscosity, which can result in increased platelet counts and plasma concentration which may stimulate thrombosis [25]. Further, the breathing of cold air may provoke pulmonary neurogenic reflexes owing to which vulnerability to arrhythmia and atherothrombosis increases; besides, pre-existing pulmonary conditions can be exacerbated by inhalation of cold air [5]. However, literature is lacking regarding the triggering temperature level.

Some of the studies have reported the association and role of atmospheric pressure in AMI; on the contrary, in our study atmospheric pressure failed to show significance [10,14]. Relative humidity and temperature variation and the difference between the maximum and minimum temperature for the day were found to be positively related to the number of primary PCI procedures; however, relative humidity (%) was insignificant in the subgroup analysis of the cold and warm seasons. Honda et al. listed humidity as a potential metrological parameter that influences the onset of AMI [14].

However, the low coefficient of determination, especially for the cold season model, indicates collective insufficiency of average temperature, humidity, and temperature variation in explaining the daily number of primary PCI procedures. It is important to understand other causes of temperature variation precipitated AMI. Increased incidence of AMI in winter can be influenced by multiple factors, which are more prevalent in winter, such as influenza, respiratory tract infections, and other seasonal behavioral patterns, such as dietary changes, physical inactiveness, and depression [26-27].

Although climatic conditions of Karachi are not as harsh as polar or non-equatorial regions, climatic changes in winter are found to be a potentially modifiable risk factor. Climatic factors also need to be addressed while adopting AMI preventive strategies.

The NICVD, Karachi, Pakistan is the largest cardiac care center in Pakistan. It not only serves the inhabitants of Karachi city but also has a significant patient flow from across Pakistan, especially suburbs and rural areas of Sindh. In our analysis, the number of primary PCI procedures at the NICVD is not explicit for the inhabitants of Karachi, while the metrological data is explicitly for Karachi city. This discrepancy in the data is the primary limitation of this study. Weather conditions in the Karachi region are very much different from other parts of the country; therefore, the study findings are limited to the Karachi region or the regions with Karachi like weather conditions.

## Conclusions

The average daily temperature was found to be negatively related to the number of primary PCIs, while humidity and daily temperature variation had a positive impact. Subgroup analysis revealed that the average daily temperature had a significant negative relationship with the number of primary PCIs in the cold season; however, no such impact was observed in the warm season.
